# Third Follow-Up of the Study on Occupational Allergy Risks (SOLAR III) in Germany: Design, Methods, and Initial Data Analysis

**DOI:** 10.3389/fpubh.2021.591717

**Published:** 2021-03-04

**Authors:** Felix Forster, Sylvia Kreißl, Laura Wengenroth, Christian Vogelberg, Erika von Mutius, Bianca Schaub, Dennis Nowak, Tobias Weinmann, Katja Radon, Jessica Gerlich

**Affiliations:** ^1^Institute and Clinic for Occupational, Social and Environmental Medicine, University Hospital, Ludwig Maximilian University Munich, Munich, Germany; ^2^Comprehensive Pneumology Centre Munich, German Centre for Lung Research, Munich, Germany; ^3^Paediatric Department, University Hospital Carl Gustav Carus Dresden, Technical University Dresden, Dresden, Germany; ^4^Dr. von Hauner Children's Hospital, University Hospital, Ludwig Maximilian University Munich, Munich, Germany

**Keywords:** asthma, occupational asthma, atopic dermatitis, rhinitis, epidemiological methods, cohort study

## Abstract

**Introduction:** Asthma and allergies are complex diseases affected by genetic and environmental factors, such as occupational and psychosocial factors, as well as interactions between them. Although childhood is a critical phase in the development of asthma and allergies, few cohort studies on occupational outcomes followed up participants from childhood onwards. We present design, methods, and initial data analysis for the third follow-up of SOLAR (Study on Occupational Allergy Risks), a prospective and population-based German asthma and allergy cohort.

**Methods:** The SOLAR cohort was initially recruited in 1995–1996 for Phase II of the German branch of the International Study of Asthma and Allergies in Childhood (ISAAC II) and followed up three times since, in 2002–2003, 2007–2009, and 2017–2018. During the third follow-up (SOLAR III), participants were between 29 and 34 years old. Since SOLAR focuses on occupational exposures, follow-ups were conducted at important points in time of the development of participants' career. To evaluate the potential of selection bias, responders and non-responders were compared based on variables from earlier study phases. In responders, frequency and pattern of missing values were examined and compared within the subsets of paper and online versions of the used questionnaires.

**Results:** In total, 1,359 participants completed the questionnaire of the third follow-up (47.3% of eligible participants). Initially, the cohort started with 6,399 participants from the ISAAC II questionnaire study. A selection process led to a study population that is more female, higher educated, smokes less and has a higher proportion of certain asthma and allergy symptoms (also in their parents) than the initial cohort. Pattern and frequency of missing values were different for paper and online questionnaires.

**Discussion:** The third follow-up of the SOLAR cohort offers the opportunity to analyze the course of asthma and allergies and their associations to environmental, occupational and psychosocial risk factors over more than 20 years from childhood to adulthood. Selection processes within the cohort might lead to bias that needs to be considered in future analyses.

## Introduction

Asthma and allergies are complex diseases affected by environmental and genetic factors as well as interactions between them (1, 2). In addition, different phenotypes of asthma have been established, based for example on the time of onset. One important type of adult-onset asthma is work-related asthma, which is associated with workplace exposures (3). So far only few cohort studies on occupational outcomes follow up participants from childhood onwards. Nevertheless, the inclusion of childhood is important since it is a critical phase in the development of asthma and allergies and because childhood symptoms might affect later job choices (4). To investigate the course of asthma and allergies from childhood to adulthood elucidating especially the role of occupational risk factors, the SOLAR study (Study on Occupational Allergy Risks) was established based on the German part of the International Study of Asthma and Allergies in Childhood Phase II (ISAAC II). Three follow-up studies have been conducted since, with a total follow-up time of more than 20 years.

The third follow-up of the Study on Occupational Allergy Risks (SOLAR III) aims to:

- further investigate the course of asthma and allergies from childhood to adulthood;
- continue the collection of data on occupational, environmental and psychosocial risk factors and investigate associations with asthma and allergies;
- study risk factors in relation to participants' age.

This article presents design and methods of SOLAR III and reports processes and results from its initial data analysis (IDA). IDA is an essential part of the study process within the conduction of observational studies. It connects data collection and analysis including the set-up of metadata, data cleaning, and data screening. IDA is necessary to obtain an analyzable data set and to identify aspects that influence interpretation and future analyses (5).

## Methods

### Study Design

The SOLAR cohort was initially recruited in 1995–1996 for Phase II of the German branch of the International Study of Asthma and Allergies in Childhood (ISAAC II). ISAAC II aimed to find potential determinants for asthma and allergy occurrence and severity around the world (6). For this, community-based random samples of children aged 9–11 years were drawn in the two study centers Munich and Dresden. An additional goal of the German branch was to investigate differences in asthma and allergies between east (Dresden) and west (Munich) of the recently reunified Germany (7). In total, 7,498 children were invited to participate and fill in a questionnaire. For both study centers, 6,399 children and their parents participated (85.3%). A random subset of children (*n* = 4,018) was also invited to clinical examinations including spirometry, tests for bronchial hyperresponsiveness using nebulized hypertonic saline, skin prick tests, specific IgE tests in blood serum, and standardized skin examinations.

In 2002–2003, the first phase of SOLAR (SOLAR I) followed-up the initial German ISAAC II cohort. Of 4,893 invited adolescents aged 16–18 years who could be re-contacted, 3,785 (77.4%) completed the questionnaire and agreed to link the data with the information from ISAAC II. Additionally, 3,053 participants (62.4%) agreed to be re-contacted for subsequent studies. In 2007–2009, 2,051 participants (70.6% of the eligible 2,904 participants) aged 19–24 years filled in the questionnaire for the second follow-up (SOLAR II). SOLAR II also included clinical examinations, comprising e.g., physical examinations, skin prick tests, and spirometry (8).

**Figure 1:**
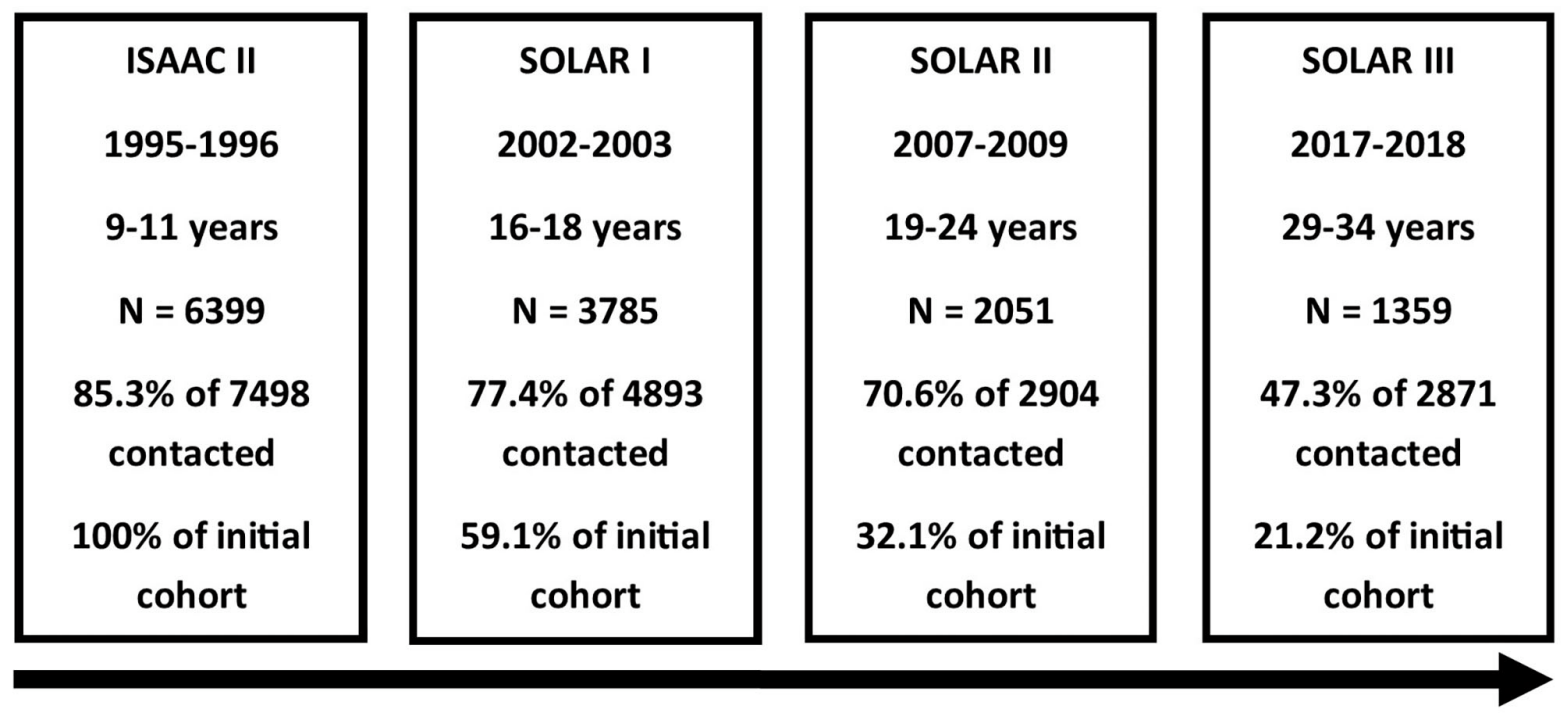
Flowchart of the SOLAR study phases. From top to bottom: study phase, time period of data collection, age of participants, number of participants, response based on number of invited participants, and proportion of ISAAC II cohort remaining.

All participants who agreed to be re-contacted in SOLAR I and for whom either an e-mail or postal address was available were invited to complete a questionnaire for the third follow-up (SOLAR III), which means that cohort members were also asked to participate in SOLAR III if they did not participate in SOLAR II without actively refusing re-contact. No clinical examinations were conducted in the third follow-up. During the field phase in 2017–2018, the participants were between 29 and 34 years old. In total, 1,359 participants completed the questionnaire (Figure 1).

All study phases were approved by the Ethical Committees of the Medical Faculty of the University of Dresden and the Bavarian Chamber of Physicians. Written informed consent, also for linking data from all study phases, was obtained from all participants (SOLAR I to III) and their legal guardians (ISAAC II, SOLAR I).

### Questionnaire Instruments

The SOLAR III questionnaire (121 items) included validated questions on:

- socio-demographics (six items)
- respiratory symptoms and disease (including asthma and wheeze) (15 items)
- rhinoconjunctivitis and hay fever (7 items)
- atopic dermatitis and hand eczema (13 items)
- domestic exposures, use of skin care products, use of disinfectants (14 items)
- smoking, exposure to environmental tobacco smoke (13 items)
- occupation (19 items)
  ◦ level of education and job type
  ◦ job history for all jobs held for at least 1 month and for at least 8 h a week
  ◦ occupational diseases and risk factors
- physical activity and use of entertainment electronics (5 items)
- body height and weight (2 items)
- use of oral contraceptives, number of pregnancies (3 items)
- depression (PHQ-2) (2 items) (9)
- work-related stress [TICS (Trier Inventory of the Assessment of Chronic Stress)] (22 items) (10, 11).

Throughout the study phases, the same questions on respiratory symptoms and disease as well as on atopic dermatitis and hand eczema were used. Those questions were originally in English and translated with back-translation into German for ISAAC (6). Questions on exposures and other variables were also kept as similar as possible throughout the study phases and came for example from the ECRHS (12) and the GA^2^LEN survey (13). Compared to the second follow-up, questions on water pipe and electronic cigarette use (water pipe questions were modified for electronic cigarettes) (14), discrimination and harassment at work (15), working conditions (16), and depression screening (9) were added in SOLAR III. Questions on job choice, accidents involving steam, gas, or smoke, state of residence, glove material, and frequency of washing hands that were still in the second follow-up questionnaire were left out in phase III. Some questions on symptoms of asthma and rhinitis were no longer kept either in order to keep the length of the questionnaire acceptable for participants. Removed questions were either not relevant anymore because of participants' age or had many missing values in earlier study phases. The questionnaire used is available as Supplementary Material.

After assessment of face validity, the content validity of the newly added questions were evaluated in a pre-test. The seven pre-test participants were sampled based on convenience and were of both genders, between 27 and 35 years old, and had low to high level of education to represent the demographic characteristics of participants (17). They were no participants of ISAAC or SOLAR and were asked to explain the presented questions to the investigator. In case of difficulties understanding the meaning of the questions, the questionnaire was revised accordingly before the pilot study.

We additionally offered the possibility to complete the questionnaire online. The open source software LimeSurvey (LimeSurvey GmbH, Hamburg, Germany) was used for setting up the online version. The survey was hosted on servers of the University Hospital, LMU Munich (Munich, Germany) to ensure data protection.

### Recruitment Methods

A pilot study including 25 participants from each study center indicated that the planned recruitment processes (e-mail and mail) worked out well. Participants for whom an e-mail address was available were contacted via e-mail with study information and were invited to fill in the online questionnaire. The remaining participants received a letter including the paper questionnaire, an informed consent form, study information, and an envelope for sending the questionnaire back free of charge. In order to ensure written informed consent as requested by the data protection representative, all participants of the online questionnaire had to print-out and send-in the signed written consent form by fax, e-mail, or postal mail. Participants were reminded twice, firstly 1 week after the initial contact and secondly one (e-mail) or two (mail) weeks later. Letters were sent out on Thursdays and, e-mails on Fridays to ensure that participants received the questionnaire toward the weekend. Because a substantial proportion of participants already had children, school holidays were avoided for the contact phase. As an incentive, participants who completed the questionnaire had the chance of winning one of ten 200€ shopping vouchers.

When e-mail addresses were invalid or e-mail invitations remained unanswered, the participants were re-contacted via postal mail. When postal addresses were outdated, the local population registries were asked for the current address. Additionally, participants without informed consent form (mainly online participation, 85.8% in study center Munich and 98.4% in Dresden) were reminded via postal mail and, if no response was registered after 21 days, by telephone. The letter contained a ready-to-sign consent form and a post-paid envelope. Thereby, 92.0% (Munich) and 83.5% (Dresden) of the missing forms were received.

### Data Processing and Cleaning

Paper questionnaires were entered manually by two independent staff members. Differences between both entries were compared to the paper questionnaire and changed accordingly. Every change was documented to assure the possibility of replication. Concordant entries were assumed correct. Missing values were coded either “missing” or “not applicable” depending on what applied.

Plausibility checks were conducted to obtain a dataset as error-free as possible. Questions filtering subsequent questions were checked for plausible values. If plain text answers contained options that were selectable in the corresponding single or multiple-choice questions, these options were assigned.

Job histories were coded manually by two independent staff members according to the International Standard Classification of Occupations 88 (ISCO-88) classification (18). Afterwards, differing codes were compared in an expert re-evaluation step. Exposure to potential occupational risk factors for asthma and allergies was assessed by linking exposure profiles from the asthma-specific job-exposure-matrix (JEM) by Le Moual and colleagues (19) with the ISCO-88 codes.

All steps of crude data processing and cleaning were documented either in R software (20) scripts, tables, or the data dictionary. This ensures that the cleaned, final dataset can be reproduced from the original variables.

### Data Screening and Evaluation of Selection Bias

In order to identify relevant aspects that influence interpretation and future analysis (5), frequency and pattern of missing values were examined and compared within the subsets of answers given by paper and online questionnaires.

To evaluate the potential of selection bias, responders and non-responders were compared in two different ways: First, all SOLAR III responders were compared with ISAAC II participants not responding in SOLAR III with regard to sex, parental history of asthma, parental history of asthma or allergies, and parental socio-economic status (SES). These variables were measured in ISAAC II. Second, all SOLAR III responders were compared with SOLAR I participants not responding in SOLAR III in terms of the outcomes 12-months prevalence of wheezing, asthma, allergic rhinitis, and atopic dermatitis, life-time prevalence of doctor diagnosed asthma, participants own SES, smoking, physical activity (PA), work-related stress, and occupational exposure to potential occupational risk factors for asthma and allergies measured at SOLAR I. SOLAR I results were considered rather than SOLAR II results as they included a larger number of SOLAR III non-respondents.

Parental history of asthma was defined as present if at least one parent reported ever having had asthma. Parental history of asthma or allergies was defined as present if at least one parent reported ever having had asthma, hay fever, or dermatitis. Parental as well as participant's SES were considered high for 12 or more years of education (for at least one parent for parental SES). Twelve-months prevalence of asthma was defined as symptoms of wheezing within the last 12 months prior to the survey and a doctor diagnosis of asthma or multiple doctor diagnoses of asthmatic bronchitis (7). Twelve-months prevalence of allergic rhinitis was defined as having problems with sneezing or a runny blocked nose without having a cold during the last 12 months that were accompanied by itchy-watery eyes. Twelve-months prevalence of atopic dermatitis was defined as ever having had eczema for at least 6 months with symptoms during the 12 months prior to study and the itchy rash at any time affecting any of the following places: the folds of the elbows, behind the knees, in front of the ankles, in the face, or around the neck (21). Participants were defined as smokers if they smoked at least 20 packs in their life or at least one cigarette per day or one cigar per week for 1 year (22). PA was classified as no PA (never doing physical exercise), low PA (physical exercise between less than once a month and once a week), and high PA (physical exercise more than once a week). Work-related stress was measured by the TICS (10, 11). The items of two scales, work overload and work discontent, were summed up separately and translated to an age-specific *T*-value. For each scale, a binary variable was created which was defined as positive if the *T*-value and its 95% confidence interval exceeded the value of 50 (10). Occupational exposure to potential occupational risk factors for asthma and allergies was defined as present if the participant ever had a job that was linked to a relevant exposure by an asthma-specific job-exposure-matrix (23).

## Results

### Response

In total, 3,053 participants, who agreed to be re-contacted in the first follow-up, were asked to participate in the SOLAR III study (Table 1). Of those, 153 could not be contacted because of missing e-mail and postal addresses, 15 had died, and 14 had actively refused to be re-contacted. Of the remaining 2,871 SOLAR I participants, 1,359 answered the questionnaire (47% of the eligible sample). Response was considerably higher in the study center Dresden (56%) compared to Munich (39%). Of the 1,359 participants in SOLAR III, 216 had not participated in SOLAR II (22% of SOLAR II non-responders).

**Table 1:** Participation in the SOLAR study phases.

	**Total**	**Munich**	**Dresden**
	***n* (%)**	***n* (%)**	***n* (%)**
**ISAAC Phase II (Questionnaire** **study)**	6,399 (85.3)^a^	3,354 (87.6)	3,045 (83.0)
**SOLAR I**	3,785 (77.4)^b^	2,043 (81.5)	1,742 (73.0)
Agreed to be re-contacted	3,053 (80.7)	1,534 (75.1)	1,519 (87.2)
**SOLAR II**	2,051 (70.6)^c^	1,008 (69.6)	1,043 (71.1)
**SOLAR III**			
Contacted	3,053 (100.0)	1,534 (100.0)	1,519 (100.0)
**Lost participants**	182 (6.0)	46 (3.0)	136 (9.0)
No valid address available	153 (5.0)	33 (2.2)	120 (7.9)
Deceased	15 (0.5)	6 (0.4)	9 (0.6)
Participant refused further contact	14 (0.5)	7 (0.5)	7 (0.5)
**Eligible sample**	2,871 (94.0)	1,488 (97.0)	1,383 (91.0)
**Response**	1,359 (47.3)^d^	585 (39.3)	774 (56.0)
of these			
Participation in SOLAR II	1,143 (84.1)	496 (84.8)	647 (83.6)
No participation in SOLAR II	216 (15.9)	89 (15.2)	127 (16.4)
Online questionnaire	787 (57.9)	323 (55.2)	464 (59.9)
Paper questionnaire	572 (42.1)	262 (44.8)	310 (40.1)

a *6,399 of 7,498 invited children*.

b *3,785 of 4,893 invited adolescents who could be re-contacted*.

c *2,051 of 2,904 invited adults who could be re-contacted*.

d *1,359 of 2,871 eligible participants*.

### Non-participation

A higher proportion of SOLAR III participants was female (61 vs. 47%) and had a high parental SES (59 vs. 46%) compared to ISAAC II participants not participating in SOLAR III (Table 2). While no difference was found for parental history of asthma, a higher proportion of SOLAR III participants had parents with a history of asthma or allergies (46 vs. 39%).

**Table 2:** Non-responder-analysis comparing all SOLAR III participants to ISAAC II participants not responding in SOLAR III based on baseline data.

	**Responders SOLAR III** ***N* = 1,359**		**ISAAC II participants not responding in SOLAR III** ***N* = 5,040**	
	**Available responses** ***n* (%)**	**% (95%-CI)**	**Available responses** ***n* (%)**	**% (95%-CI)**
Female	1,359 (100.0)	60.5 (57.9–63.1)	5,036 (99.9)	46.5 (45.1–47.9)
Parental history of asthma^a^	1,243 (91.5)	9.5 (7.9–11.1)	4,466 (88.6)	9.9 (9.0–10.8)
Parental history of asthma or allergies^b^	1,346 (99.0)	46.0 (43.3–48.7)	4,932 (97.9)	39.3 (37.9–40.7)
Parental SES (high)^c^	1,338 (98.5)	59.3 (56.7–61.9)	4,789 (95.0)	45.8 (44.4–47.2)

a *At least one parent reported ever having had asthma*.

b *At least one parent reported ever having had asthma or hay fever or dermatitis*.

c *12 or more years of education for at least one parent*.

Compared to SOLAR I participants not participating in SOLAR III, participants' SES was also higher at SOLAR I (60 vs. 44%). In addition, during SOLAR I, SOLAR III participants were more likely to report symptoms of atopic dermatitis than SOLAR I participants not responding in SOLAR III (11 vs. 8%), and less likely to be ever smokers (29 vs. 38%). No differences were seen for the other variables under study (Table 3).

**Table 3:** Non-responder-analysis comparing all SOLAR III participants to SOLAR I participants not responding in SOLAR III based on SOLAR I characteristics.

	**Responders SOLAR III** ***N* = 1,359**		**SOLAR I participants not responding in SOLAR III *N* = 2,570^a^**	
	**Available responses *n* (%)**	**% (95%-CI)**	**Available responses *n* (%)**	**% (95%-CI)**
Symptoms of wheezing within the last 12 months	1,354 (99.6)	14.7 (12.8–16.6)	2,547 (99.1)	15.0 (13.6–16.4)
Doctor diagnosis of asthma	1,334 (98.2)	7.2 (5.8–8.6)	2,515 (97.9)	8.1 (7.0–9.2)
12-months prevalence of asthma^b^	1,346 (99.0)	4.2 (3.1–5.3)	2,534 (98.6)	5.2 (4.3–6.1)
12-months prevalence of allergic rhinitis^c^	1,342 (98.7)	22.4 (20.2–24.6)	2,529 (98.4)	22.1 (20.5–23.7)
12-months prevalence of atopic dermatitis^d^	1,345 (99.0)	10.9 (9.2–12.6)	2,529 (98.4)	7.6 (6.6–8.6)
Participant's SES (high)^e^	1,351 (99.4)	59.5 (56.9–62.1)	2,548 (99.1)	44.1 (42.2–46.0)
Smoking^f^	1,346 (99.0)	29.1 (26.7–31.5)	2,546 (99.1)	37.9 (36.0–39.8)
Physical activity (high)^g^	1,353 (99.6)	50.3 (47.6–53.0)	2,554 (99.4)	48.9 (47.0–50.8)
Physical activity (low)^h^		44.3 (41.7–46.9)		42.2 (40.3–44.1)
Work overload^i^	1,348 (99.2)	27.7 (25.3–30.1)	2,516 (97.9)	25.7 (24.0–27.4)
Work discontent^i^	1,347 (99.1)	46.2 (43.5–48.9)	2,516 (97.9)	49.2 (47.2–51.2)
Exposure to any potential occupational risk factors for asthma and allergies^j^	1,335 (98.2)	14.2 (12.3–16.1)	2,450 (95.3)	13.8 (12.4–15.2)

a *The analysis for this table is based on all 3,929 SOLAR I participants including those, who did not give consent for linking the data to data from other study phases*.

b *Symptoms of wheezing within the last 12 months and a doctor diagnosis of asthma or multiple doctor diagnoses of asthmatic bronchitis*.

c *Having problems with sneezing or a runny blocked nose without having a cold during the last 12 months that were accompanied by itchy-watery eyes*.

d *Ever having had eczema for at least 6 months with symptoms during the 12 months prior to study and the itchy rash at any time affecting any of the following places: the folds of the elbows; behind the knees; in front of the ankles; under the buttocks; or around the neck, ears, or eyes*.

e *12 or more years of education*.

f *Smoked at least 20 packs in their life or for a year at least one cigarette per day or one cigar per week*.

g *Physical exercise more than once a week*.

h *Physical exercise between less than once a month and once a week*.

i *Age-specific T-value of item sum of corresponding scale and its 95% confidence interval exceeded the value of 50*.

j *Ever having had a job that was linked to a relevant exposure by an asthma-specific job-exposure-matrix*.

### Missing Data Pattern

In the total SOLAR III dataset, 3% of values were missing. Questions with the highest proportion of missing values were on environmental tobacco smoke (ETS) (11%), quitting jobs because of symptoms of asthma or allergies (6%), doctor diagnosis of respiratory outcomes (6%), skin-straining activities at home, including cleaning without gloves, construction or renovation, gardening or farming, or other tasks that could be straining for the skin due to wet conditions, chemicals or other factors (6%), wheezing (6%) or symptoms of rhinoconjunctivitis (5%) due to an occupation, and duration of glove use (5%).

**Figure 2:**
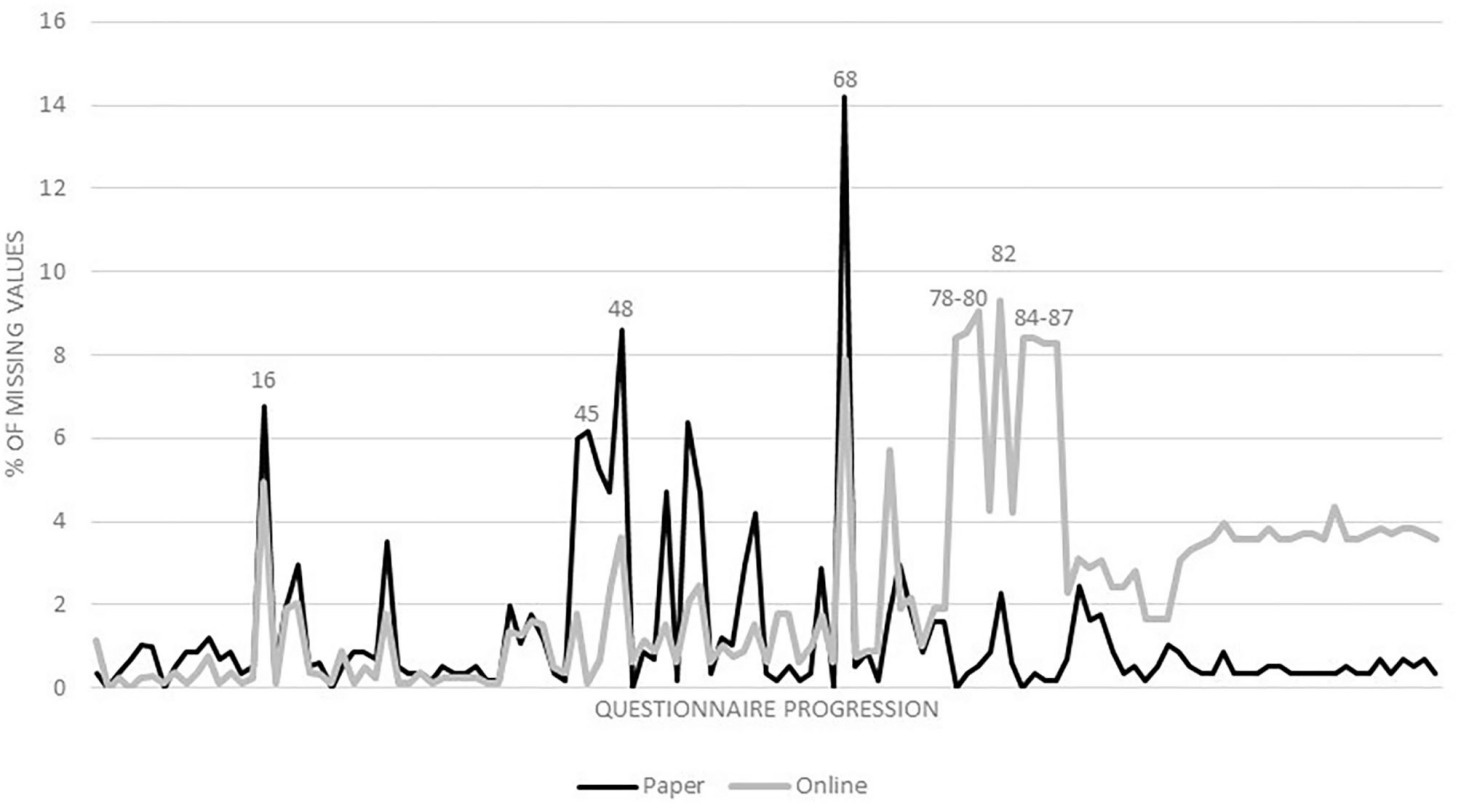
Percentage of missing values for paper and online questionnaires from first to last questionnaire item. Question numbers: 16: doctor diagnosis of respiratory outcomes; 45: indoor mold; 48: skin-straining activities at home; 68: ETS; 78–79: symptoms of rhinoconjunctivitis due to an occupation; 80: wheezing due to an occupation; 82: quitting jobs because of symptoms of asthma or allergies; 84: use of gloves; 85: duration of glove use; 86: declaration of occupational disease; 87: reason of declaration of occupational disease.

Generally speaking, online questionnaires had lower proportions of missing values in the first half of the questionnaire, while paper questionnaires had lower proportions of missing values in the second half (Figure 2). Questions with the highest difference in the proportions of missing values were on wheezing (9%-points) or symptoms of rhinoconjunctivitis (8%-points) due to an occupation, use of gloves (8%-points), including duration (8%-points), declaration of occupational disease, including its reason (8%-points), with a lower proportion of missing values for paper questionnaires, and on ETS (6%-points) and indoor mold (6%-points) with a lower proportion of missing values for online questionnaires.

## Discussion

We present design, methods, and results from the initial data analysis for the third follow-up of a German prospective and population-based asthma and allergy cohort. SOLAR started with the German ISAAC II participants in two study centers. We followed this cohort for more than 20 years from elementary school until the early thirties. The follow-ups were placed at important points in time of the participant's career: around the transition from school to work or university, around the transition from university to work, and after being settled in working life. Because of the long follow-up time, the study offers the opportunity to link (occupational) information from adulthood to data from childhood.

In the presented follow-up, no clinical examinations were feasible. Although examinations might decrease errors for example in asthma measurement, it would have negatively affected the feasibility of the study and probably also the willingness of cohort members to further participate. In addition to the initial examination in the ISAAC II study phase, an examination was conducted in the second follow-up when participants had already reached adulthood. Back then, only 40% of the eligible study population participated in the clinical part (8). Because validated questions were used throughout the study, we came to the conclusion that accuracy is maximized best by focusing on reaching a high response in the questionnaire study.

Many cohort studies investigating work-related asthma recruited workers from a specific occupation to investigate effects of a certain exposure. Often these cohorts had a few hundred participants and were followed for a time period between a few months and several years (24). Usually, eligible workers were either already exposed for a certain time or enrolled at the beginning of their job. To focus on new and therefore unexposed workers, some cohorts recruited apprentices and followed them during their training (25). Other cohorts focused on estimating asthma incidences attributable to workplace exposures (26, 27). In contrast to the mentioned studies, SOLAR tries to investigate the course of asthma and allergies, including work-related phenotypes, from childhood to adulthood.

Although the initial cohort was population-based, a selection process led to a study population that is more female, higher educated, smokes less, and has a higher proportion of people with atopic dermatitis at the end of childhood. The proportion of participants with at least one parent that reported ever having had asthma, hay fever, or dermatitis was increased as well. The selection process was already present in earlier follow-ups (8). In the initial ISAAC cohort, however, participants and non-participants of clinical examinations were similar regarding atopic diseases in children and parents, parental education, family size, passive smoke exposure, and sex (28). Regarding the 1,099 invited children that didn't participate at all, no information on potential selection was available. It implies the potential of selection bias that needs to be considered carefully in analyses of follow-up data. Depending on the research question, the available information will be used to obtain less biased results, e.g., by adjusting estimates or multiply imputing missing values.

Since the cohort underwent a selection process over the years of follow-up, the generalizability of the study's results might be limited if selection bias affects the internal validity of the study. However, since the study's goal is the investigation of associations between occupational, environmental, and psychosocial exposures and asthma and allergy outcomes, this selection process does not affect the generalization of results on the basic association to other populations as long as the internal validity is not substantially affected. Nevertheless, asthma and allergies are complex diseases for which reason associations might vary for different genotypes, age groups and exposure histories. Therefore, genetic background and age of participants as well as environmental factors that might interact need to be considered when generalizing the results of the SOLAR study to other populations. After all, comparisons of future results to other cohorts is necessary for drawing conclusions about associations.

A strength of the SOLAR study is its still relatively high sample size after more than two decades of follow-up. This response could be reached using several methods to increase participation, including incentives, e-mail, postal, and telephone reminders as well as envelopes for returning study documents free of charge. Since e-mail addresses were collected in earlier study phases for a substantial part of the cohort, a valid postal address was not necessary for reaching these participants. An online version of the questionnaire was used to simplify participation for individuals with known e-mail addresses. One drawback of the online version was the difficulty to get informed consent, since it was necessary for the participants to conduct an extra step of printing and sending the signed consent form. The number of missing forms and therefore of excluded questionnaires could be reduced substantially by sending out postal reminders, which made it necessary to get a valid postal address for some of the participants with known e-mail addresses after all.

The questions with higher proportions of missing values in the subset of online questionnaires mentioned earlier were all asked in the second half of the questionnaire. This might indicate that some participants quit before finishing and that 121 items are therefore too many for an online questionnaire. An alternative explanation for these differences might be that the questionnaire was too long in general and that we just received more incomplete online questionnaires than incomplete paper questionnaires as those were not sent-in.

In general, the online questionnaire was a good addition, because including logical links that made it possible to skip questions that were not applicable, and making it mandatory for continuing to answer certain questions, led to less missing values than in the paper version for most questions in the first half of the questionnaire. Apart from that, the use of online questionnaires saved time (of participants and the research team) and money for sending invitations and data entry. Although the proportion of missing values is not too high in total, multiple imputation methods should be used to limit potential biases. The information on the type of questionnaire (paper vs. online) should be included in the imputation process since it is a potential cause or correlate of missingness (29).

In conclusion, the third follow-up of the SOLAR cohort offers the opportunity to analyze the course and risk factors of asthma and allergies over more than 20 years from childhood to adulthood. The focus on the occupational environment, including the participants' full job histories, makes it possible to investigate occupational exposures in particular. The use of online questionnaires contributed to the feasibility of conducting a third follow-up and still yielding an adequate size of the study population. However, selection processes within the cohort might lead to sources of bias that need to be considered in future analyses.

## Data Availability Statement

The datasets presented in this article are not readily available because of data protection reasons. Requests to access the datasets should be directed to the corresponding author.

## Ethics Statement

The studies involving human participants were reviewed and approved by the Ethical Committees of the Medical Faculty of the University of Dresden (EK 163042015) and the Bavarian Chamber of Physicians (mb BO 17015). The patients/participants provided their written informed consent to participate in this study.

## Author Contributions

FF drafted the manuscript. Data acquisition was coordinated by FF, SK, and LW. Data was interpreted by FF, JG, and KR. CV, JG, LW, TW, KR, DN, BS, EM, SK, and FF contributed substantially to the conception and design of the study. All authors contributed to the article and approved the submitted version.

## Conflict of Interest

EM reports grants from German Ministry of Education and Research, during the conduct of the study; personal fees from Massachassuetts Medical Society, personal fees from American Academy of Allergy, Asthma and Immunology, personal fees from Novartis Pharma SAS, personal fees from PharmaVentures, personal fees from OM Pharma, personal fees from Decision Resources, personal fees from The Chinese University of Hongkong, personal fees from University of Copenhagen, personal fees from HAL Allergie GmbH, personal fees from Ökosoziales Forum Oberösterreich, personal fees from Mundipharma, personal fees from American Thoracic Society, personal fees from AbbVie Deutschland GmbH & Co. KG, personal fees from University of Tampere, personal fees from European Commission, personal fees from University of Turku, personal fees from University Helsinki, personal fees from Peptinnovate, outside the submitted work. The remaining authors declare that the research was conducted in the absence of any commercial or financial relationships that could be construed as a potential conflict of interest.

## Abbreviations

ETS: Environmental tobacco smoke
IDA: Initial data analysis
ISAAC: International Study of Asthma and Allergies in Childhood
ISCO: International Standard Classification of Occupations
JEM: Job-exposure-matrix
PA: Physical activity
SES: Socio-economic status
SOLAR: Study on Occupational Allergy Risks
TICS: Trier Inventory of the Assessment of Chronic Stress.
