# Supplementary material for: Third Follow-Up of the Study on Occupational Allergy Risks (SOLAR III) in Germany: Design, Methods, and Initial Data Analysis
Source: Front Public Health. 2021 Mar 4;9:591717. doi: 10.3389/fpubh.2021.591717 (PMC7969718; doi:10.3389/fpubh.2021.591717)
Supplement: Supplementary file 1 [file Data_Sheet_1.PDF]

SOLAR III Questionnaire

Original questionnaire in German (below). Questionnaire was only translated to English for presentation in this additional file. When German questions were based on existing English ones, the original wording was used. All other questions were translated by one person without back-translating and shouldn't, therefore, be used without further validation.

English Version

General information

1. **When were you born?** |__|__| |__|__| 19|__|__|
   Day Month Year

2. **Are you male or female?**
   ☐ male
   ☐ female

3. **What is your marital status?**
   ☐ single
   ☐ married
   ☐ divorced
   ☐ widowed

4. **Do you have children? (Adopted and stepchildren included)**
   ☐ No
   ☐ Yes

5. **How many children do you have?** |__|__| Children

6. **In what year were your children born?**

   Child 1 Child 2 Child 3

   20|__|__| 20|__|__| 20|__|__|

Health

7. **Have you had wheezing or whistling in your chest at any time in the last 12 months?**
   ☐ No
   ☐ Yes

8. **Have you had this wheezing or whistling when you did not have a cold?**
   ☐ No
   ☐ Yes

9. **Have you had an attack of shortness of breath that came during the day when you were at rest at any time in the last 12 months?**
   - ☐ No
   - ☐ Yes

10. **Have you had an attack of shortness of breath that came on following strenuous activity at any time in the last 12 months?**
   - ☐ No
   - ☐ Yes

11. **Have you been woken by an attack of shortness of breath at any time in the last 12 months?**
   - ☐ No
   - ☐ Yes

12. **Do you have a cough on most days for as much as 3 months each year?**
   - ☐ No
   - ☐ Yes

13. **If yes, has this condition existed for at least 2 years?**
   - ☐ No
   - ☐ Yes

14. **Do you bring up phlegm from your chest on most days for as much as 3 months each year?**
   - ☐ No
   - ☐ Yes

15. **If yes, has this condition existed for at least 2 years?**
   - ☐ No
   - ☐ Yes

16. **Have you ever been diagnosed <u>by a doctor</u> with any of the following diseases?**

   Asthma
   - ☐ Never
   - ☐ Once
   - ☐ More than once

   Spastic / asthmatic bronchitis
   - ☐ Never
   - ☐ Once
   - ☐ More than once

   Chronic bronchitis
   - ☐ Never

☐ Once
☐ More than once

17. Have you ever had asthma?
☐ No
☐ Yes

18. How old were you when you had your first asthma attack? |__|__| Years

19. How old were you when you had your most recent attack of asthma? |__|__| Years

20. Have you had an attack of asthma in the last 12 months?
☐ No
☐ Yes

21. Are you currently taking any medicines, including inhalers, aerosols or tablets, for asthma?
☐ No
☐ Yes
Please specify:

22. In the past 12 months, have you had a problem with sneezing, or a runny, or blocked nose when you did not have a cold or the flu?
☐ No
☐ Yes

23. Has this nose problem been accompanied by itchy-watery eyes?
☐ No
☐ Yes

24. Do you have any nasal allergies including "hay fever"?
☐ No
☐ Yes

25. Have you had any nasal allergies including "hay fever" in the last 12 months?
☐ No
☐ Yes

26. Have you ever been diagnosed <u>by a doctor</u> with any nasal allergies including "hay fever"?
☐ No
☐ Yes

27. **How old were you when you had nasal allergies or hay fever for the first time?** |__|__| Years

28. **Have you ever been desensitized / hyposensitized against an allergy (e.g. you regularly get injections or put a tablet under your tongue)?**
    - ☐ No
    - ☐ Yes
    - ☐ I don't know

29. **Have you ever had atopic eczema?**
    - ☐ No
    - ☐ Yes

30. **Have you ever been diagnosed <u>by a doctor</u> with atopic eczema?**
    - ☐ No
    - ☐ Yes

31. **Have you ever had an itchy rash which was coming and going for at least six months?**
    - ☐ No
    - ☐ Yes

32. **Have you had this itchy rash at any time in the last 12 months?**
    - ☐ No
    - ☐ Yes

33. **Does this affect <u>only</u> your hands?**
    - ☐ No
    - ☐ Yes

34. **Has this itchy rash at any time affected any of the following places: the folds of the elbows, behind the knees, in front of the ankles, or around the neck, ears or eyes?**
    - ☐ No
    - ☐ Yes

35. **Do you have nickel allergy (e.g. earrings, jeans button, watch straps)?**
    - ☐ No
    - ☐ Yes

36. **Have you ever had eczema on your hands?**
    - ☐ No

☐ Yes

37. Have you ever had eczema on your wrists or forearms (except in the elbows)?

☐ No
☐ Yes

38. When was the last time you had eczema on your hands, wrists or forearms?

Eczema on hands

☐ I have it now
☐ Not now, but within the last 3 months
☐ 3-12 months ago
☐ More than 12 months ago
In which year for the last time? |__|__|__|__|

Eczema on wrists or forearms

☐ I have it now
☐ Not now, but within the last 3 months
☐ 3-12 months ago
☐ More than 12 months ago
In which year for the last time? |__|__|__|__|

39. Have you noticed that contact with certain materials, chemicals or anything else <u>in your workplace</u> makes your eczema worse?

Eczema on hands

☐ No
☐ Yes
Please specify:
☐ I don't know

Eczema on wrists or forearms

☐ No
☐ Yes
Please specify:
☐ I don't know

☐ I'm not working at the moment

40. Have you noticed that contact with certain materials, chemicals or anything else <u>outside your workplace</u> makes your eczema worse?

Eczema on hands

☐ No
☐ Yes
Please specify:

☐ I don't know

Eczema on wrists or forearms

☐ No

☐ Yes

Please specify:

☐ I don't know

41. Does your eczema improve when you are <u>not at your normal workplace</u> (e.g. weekends or longer periods)?

Eczema on hands

☐ No

☐ Yes, sometimes

☐ Yes, usually

☐ I don't know

Eczema on wrists or forearms

☐ No

☐ Yes, sometimes

☐ Yes, usually

☐ I don't know

☐ I'm not working at the moment

Your home

42. How many years have you lived in your present home?

|__|__| Months

or

|__|__| Years

43. What kind of floor covering is in your bedroom?

☐ Fitted carpets

☐ Loose carpets

☐ Bare floor

44. Have you made any changes in your home within the last 10 years because you had allergic problems?

☐ Changed floor covering

When (Year)? 20|__|__|

☐ Changed bedding

When (Year)? 20|__|__|

☐ Removed pets

When (Year)? 20|__|__|

☐ None of the above

45. Has there ever been mould or mildew on any surface, other than food, inside the home?

At present

☐ No
☐ Yes
☐ I don't know

Within the last 10 years

☐ No
☐ Yes
☐ I don't know

Which rooms have been affected?

Living room

☐ No mould
☐ Smaller than 0.5 m^2
☐ Between 0.5 and 1 m^2
☐ Larger than 1 m^2

Bedroom

☐ No mould
☐ Smaller than 0.5 m^2
☐ Between 0.5 and 1 m^2
☐ Larger than 1 m^2

Children's room

☐ No mould
☐ Smaller than 0.5 m^2
☐ Between 0.5 and 1 m^2
☐ Larger than 1 m^2

Kitchen

☐ No mould
☐ Smaller than 0.5 m^2
☐ Between 0.5 and 1 m^2
☐ Larger than 1 m^2

Bathroom

☐ No mould
☐ Smaller than 0.5 m^2
☐ Between 0.5 and 1 m^2
☐ Larger than 1 m^2

Other room

☐ No mould
☐ Smaller than 0.5 m^2
☐ Between 0.5 and 1 m^2
☐ Larger than 1 m^2

46. Do you have contact with any of the following animals?
Inside your home
☐ No animals
☐ Dog
☐ Cat
☐ Bird
☐ Other animals
Please specify:
In any other home (at least once a month)
☐ No animals
☐ Dog
☐ Cat
☐ Bird
☐ Other animals
Please specify:

47. Which of your pets are allowed into your bedroom?
☐ None
☐ Dog
☐ Cat
☐ Other animals
Please specify:

48. How often are you involved in the following activities outside your job?
Cleaning / Dishwashing without gloves
☐ Never
☐ < 1 day per week
☐ 1-3 days per week
☐ ≥ 4 days per week
House building / renovation
☐ Never
☐ < 1 day per week
☐ 1-3 days per week
☐ ≥ 4 days per week
Garden / Agriculture

☐ Never
☐ < 1 day per week
☐ 1-3 days per week
☐ ≥ 4 days per week

Other activities that could be harmful to the skin due to wetness, chemicals or other factors

☐ Never
☐ < 1 day per week
☐ 1-3 days per week
☐ ≥ 4 days per week

Please specify:

49. Do you use skin protection / skin care products?

☐ No
☐ Yes

50. How often do you use them at home?

About |__|__| times a day
or
About |__|__| times a week

51. How often do you use them at work?

About |__|__| times a day
or
About |__|__| times a week
☐ I'm not working at the moment

52. On how many days per week do you use the following sprays?

Furniture sprays

☐ Never
☐ < 1 day per week
☐ 1-3 days per week
☐ ≥ 4 days per week

Glass cleaning sprays (window, mirror)

☐ Never
☐ < 1 day per week
☐ 1-3 days per week
☐ ≥ 4 days per week

Sprays for carpets or curtains

☐ Never

☐ < 1 day per week
☐ 1-3 days per week
☐ ≥ 4 days per week

Oven sprays

☐ Never
☐ < 1 day per week
☐ 1-3 days per week
☐ ≥ 4 days per week

Ironing sprays

☐ Never
☐ < 1 day per week
☐ 1-3 days per week
☐ ≥ 4 days per week

Air freshener sprays

☐ Never
☐ < 1 day per week
☐ 1-3 days per week
☐ ≥ 4 days per week

Deodorant sprays

☐ Never
☐ < 1 day per week
☐ 1-3 days per week
☐ ≥ 4 days per week

Other sprays

☐ Never
☐ < 1 day per week
☐ 1-3 days per week
☐ ≥ 4 days per week
Please specify:

53. Do you use disinfectants?

☐ No
☐ Yes

54. How many hours per day do you use these disinfectants?

At home

☐ Never
☐ < 1 hours per day
☐ 1-3 hours per day

☐ ≥ 4 hours per day

At work

☐ Never

☐ < 1 hours per day

☐ 1-3 hours per day

☐ ≥ 4 hours per day

☐ I'm not working at the moment

55. On how many days per week (at work or at home) do you use the following disinfection methods?

Spray disinfection

☐ Never

☐ < 1 day per week

☐ 1-3 days per week

☐ ≥ 4 days per week

Wash off by hand with disinfectants

☐ Never

☐ < 1 day per week

☐ 1-3 days per week

☐ ≥ 4 days per week

Disinfect with a machine

☐ Never

☐ < 1 day per week

☐ 1-3 days per week

☐ ≥ 4 days per week

Clean surfaces with sponge / cloth and disinfectant

☐ Never

☐ < 1 day per week

☐ 1-3 days per week

☐ ≥ 4 days per week

Wiping or scrubbing floors with disinfectants

☐ Never

☐ < 1 day per week

☐ 1-3 days per week

☐ ≥ 4 days per week

Other methods

☐ Never

☐ < 1 day per week

☐ 1-3 days per week
☐ ≥ 4 days per week
Please specify:

Smoking

56. **Have you ever smoked for as long as a year? "Yes" means at least 20 packs of cigarettes or 12 oz (360 grams) of tobacco in a lifetime, or at least one cigarette per day or one cigar a week for one year.**
    ☐ No
    ☐ Yes

57. **How old were you when you started smoking?** |__|__| Years

58. **Do you now smoke, as of one month ago?**
    ☐ No
    ☐ Yes

59. **How old were you when you stopped or cut down smoking?** |__|__| Years

60. **On average of the entire time you smoked, before you stopped or cut down, how much did you smoke?** |__|__| Cigarettes/day

61. **Have you ever smoked a water pipe or shisha?**
    ☐ No
    ☐ Yes

62. **Have you smoked a water pipe or shisha in the last 12 months?**
    ☐ No
    ☐ Yes

63. **When you think about the last 30 days: On how many days have you smoked water pipe or shisha?** On |__|__| days

64. **Have you ever smoked e-cigarette?**
    ☐ No
    ☐ Yes

65. **Have you smoked e-cigarette in the last 12 months?**
    ☐ No
    ☐ Yes

66. **If you now think about the last 30 days: How many ml of liquid or how many puffs do you smoke with the e-cigarette on average per day?**
|__|__|__| ml liquid/day

or
|__|__|__| number of puffs/day

67. **Have you been regularly exposed to tobacco smoke or e-cigarette steam in the last 12 months? “Regularly” means on most days or nights.**
☐ No
☐ Yes, only tobacco smoke
☐ Yes, only e-cigarette steam
☐ Yes, tobacco smoke and e-cigarette steam

68. **Please describe this in more detail: How many hours per day are you exposed to tobacco smoke from others in the following places?**
At home
|__|__| Hours
☐ Less than 1 hour
At work
|__|__| Hours
☐ Less than 1 hour
In bars, restaurants, cinemas or similar places
|__|__| Hours
☐ Less than 1 hour
In the car
|__|__| Hours
☐ Less than 1 hour
Other places
|__|__| Hours
☐ Less than 1 hour

Working situation

69. **Which vocational training qualification do you have?**
☐ No vocational qualification and not currently in vocational training
☐ Completed vocational training (apprenticeship) or school-based initial training
☐ Completed continuing vocational training (master craftsman, technical engineer, certified senior clerk)
☐ University of applied sciences degree

☐ University degree (Bachelor)
☐ University degree (Master, Diplom)
☐ Other vocational qualification
☐ Currently still in vocational training
☐ Student
☐ Other qualification
Please specify:

70. Are you currently…?
☐ In vocational training (apprenticeship or school-based)
☐ Full-time student
☐ Employed
☐ Civil servant
☐ Self-employed
☐ Unemployed
☐ Not working because of poor health
☐ Full-time house person
☐ Maternity leave, parental leave or temporal leave for other reasons
☐ Other
Please specify:

71. Have you done any work/internship for at least 1 month within the last 10 years?
☐ No
☐ Yes

72. What kind of jobs and/or internships have you had in the last ten years?
Every job you have done for at least 1 month is important. It doesn't matter whether you have done this job outside or at home, full-time or part-time, with or without pay or as a self-employed person (e.g. in a family business).
Please only include jobs here if you have worked at least 8 hours per week.
Please start with the last job.
☐ I have <u>not</u> been working for <u>at least 8 hours per week</u>
Job 1
Job / Occupation
Industry sector
When did you start this job?
|__|__|/|__|__|__|__| Month/year
If applicable: When did you stop this job?
|__|__|/|__|__|__|__| Month/year
How many hours per week do/did you work in this job?
|__|__| Hours

…
Job 10
Job / Occupation
Industry sector
When did you start this job?
|__|__|/|__|__|__|__| Month/year
If applicable: When did you stop this job?
|__|__|/|__|__|__|__| Month/year
How many hours per week do/did you work in this job?
|__|__| Hours

73. Have you experienced the following situations in your work over the past 12 months?

☐ Discrimination based on age
☐ Discrimination based on your origin, ethnicity or skin colour
☐ Discrimination based on your nationality
☐ Discrimination based on your gender
☐ Discrimination based on your religion
☐ Discrimination based on disability
☐ Discrimination based on sexual orientation
☐ I haven't experienced any of these situations

74. Have you been confronted with the following situations in the last 12 months while doing your work?

☐ Physical violence
☐ Sexual harassment
☐ Bullying
☐ I was not confronted with any of these situations

75. Do your working hours change regularly? If so, how long in advance do you normally know about these changes?

☐ No
☐ Yes, the same day
☐ Yes, the day before
☐ Yes, a few days in advance
☐ Yes, several days in advance
☐ Yes, several weeks in advance
☐ Other
Please specify:

76. How well can your working hours generally be reconciled with your family or social obligations outside work?

☐ Very well

☐ Well

☐ Not very well

☐ Not at all

77. In the last 12 months, how often have you worked in your free time to meet the demands of the job?

☐ Almost every day

☐ Once or twice a week

☐ Once or twice a month

☐ Rarely

☐ Never

78. Have you had a problem with sneezing, or a runny, or blocked nose when you did not have a cold or the flu due to one of your jobs in the last 10 years?

☐ No

☐ Yes

79. Has this nose problem been accompanied by itchy-watery eyes?

☐ No

☐ Yes

80. Have you had a feeling of tightness in your chest or a wheezing or whistling in your chest due to one of your jobs?

☐ No

☐ Yes

81. During which of your jobs did you get one of these conditions?

- ☐ Job 1
- ☐ Job 2
- ☐ Job 3
- ☐ Job 4
- ☐ Job 5
- ☐ Job 6
- ☐ Job 7
- ☐ Job 8
- ☐ Job 9
- ☐ Job 10

82. Have you had to give up one of your jobs because of these conditions?

- ☐ No
- ☐ Yes

83. Which jobs did you have to give up because of these conditions?

- ☐ Job 1
- ☐ Job 2
- ☐ Job 3
- ☐ Job 4
- ☐ Job 5
- ☐ Job 6
- ☐ Job 7
- ☐ Job 8
- ☐ Job 9
- ☐ Job 10

84. Do you wear gloves at work?

- ☐ No
- ☐ Sometimes
- ☐ Yes

85. How long do you wear gloves at work?

- ☐ Less than 25% of working time
- ☐ Between 25% and 50% of working time
- ☐ More than 50% of working time

86. Have you ever been declared as having an occupational disease?

☐ No
☐ Yes

87. Due to which conditions have you been declared as having an occupational disease?

☐ Due to respiratory conditions
☐ Due to skin conditions
☐ Due to other conditions
Please specify:

Sport

88. How <u>often</u> and <u>where</u> do you usually exercise so much that you get out of breath and sweat?

Outdoor sports

☐ Never
☐ Less than once a month
☐ Once a month
☐ Once a week
☐ 2-3 times a week
☐ 4-6 times a week
☐ Every day

Indoor sports

☐ Never
☐ Less than once a month
☐ Once a month
☐ Once a week
☐ 2-3 times a week
☐ 4-6 times a week
☐ Every day

89. <u>How many hours a week</u> and <u>where</u> do you usually exercise so much that you get out of breath or sweat?

Outdoor sports

☐ None
☐ About 0.5 hours
☐ About 1 hour
☐ About 2-3 hours
☐ About 4-6 hours

☐ 7 hours and more

Indoor sports

☐ None
☐ About 0.5 hours
☐ About 1 hour
☐ About 2-3 hours
☐ About 4-6 hours
☐ 7 hours and more

90. Which sports do you practice?

☐ Running, ball games, cycling, inline skating
☐ Swimming
☐ Mixed exercise (e.g. fitness studio)
☐ Other sports

Please specify:

91. How many hours do you spend in your free time...

...per day at the computer / game console / smartphone / tablet?

|__|__| Hours
☐ Less than 1 hour

...per day watching TV (also videos / DVDs / streaming of programmes)?

|__|__| Hours
☐ Less than 1 hour

92. How many hours do you spend at work...

…per day at the computer / smartphone / tablet?

|__|__| Hours
☐ Less than 1 hour

Physical development

93. What's your height? |__|__|__| cm

94. What's your weight? |__|__|__| kg

95. Are you currently taking the contraceptive pill or other hormonal contraceptives?

☐ No
☐ Yes

96. How many years have you been using the contraceptive? For about |__|__| years

97. How many times have you been pregnant?

|__| Times

☐ Never

Stress situations

<u>**Over the last 2 weeks**</u>**, how often have you been bothered by any of the following problems?**

98. **1.1 Little interest or pleasure in doing things**

☐ Not at all

☐ Several days

☐ More than half the days

☐ Nearly everyday

1.2

99. **1.3 Feeling down, depressed, or hopeless**

☐ Not at all

☐ Several days

☐ More than half the days

1.4 ☐ Nearly everyday

The following questions are to be judged with respect to the frequency of their occurrence in the last year. In each case you will be asked to indicate if the experiences and events described below occurred never, rarely, sometimes, often or very often. Please think of your life this past year ans try to remember how often the respective experience/event occurred.

100. To postpone urgently needed rest and recreation

☐ Never

☐ Rarely

☐ Sometimes

☐ Often

☐ Very often

101. Situations in which I have to make an effort to gain the trust of others

☐ Never

☐ Rarely

☐ Sometimes

☐ Often

☐ Very often

102.Too little time to execute my daily tasks

☐ Never
☐ Rarely
☐ Sometimes
☐ Often
☐ Very often

103.Satisfaction from work (school, apprenticeship, studies) that I have to perform daily

☐ Never
☐ Rarely
☐ Sometimes
☐ Often
☐ Very often

104.Situations in which I have to strive for a good relationship with others

☐ Never
☐ Rarely
☐ Sometimes
☐ Often
☐ Very often

105.Times when I have to perform tasks that I am not at all willing to do

☐ Never
☐ Rarely
☐ Sometimes
☐ Often
☐ Very often

106.I have tasks to perform in which I am under critical observation

☐ Never
☐ Rarely
☐ Sometimes
☐ Often
☐ Very often

107.Experience that what I have to do is too much for me

☐ Never

- ☐ Rarely
- ☐ Sometimes
- ☐ Often
- ☐ Very often

108. I have tasks to do where I cannot disappoint others

- ☐ Never
- ☐ Rarely
- ☐ Sometimes
- ☐ Often
- ☐ Very often

109. Contacts with other people with whom I need to leave a good impression

- ☐ Never
- ☐ Rarely
- ☐ Sometimes
- ☐ Often
- ☐ Very often

110. Being overwhelmed due to various tasks that I have to perform

- ☐ Never
- ☐ Rarely
- ☐ Sometimes
- ☐ Often
- ☐ Very often

111. Situations in which it depends entirely on me whether a contact with another person is satisfactory

- ☐ Never
- ☐ Rarely
- ☐ Sometimes
- ☐ Often
- ☐ Very often

112. I have tasks to fulfil in which I must prove myself

- ☐ Never
- ☐ Rarely
- ☐ Sometimes
- ☐ Often

☐ Very often

113. Negativity with respect to my day to day work

☐ Never
☐ Rarely
☐ Sometimes
☐ Often
☐ Very often

114. Too many responsibilities that I have to fulfil

☐ Never
☐ Rarely
☐ Sometimes
☐ Often
☐ Very often

115. Situations in which I have to make an effort to please others

☐ Never
☐ Rarely
☐ Sometimes
☐ Often
☐ Very often

116. The wish to change my current job (school, apprenticeship, studies)

☐ Never
☐ Rarely
☐ Sometimes
☐ Often
☐ Very often

117. I have to fulfil tasks that are connected with high expectations

☐ Never
☐ Rarely
☐ Sometimes
☐ Often
☐ Very often

118. Feeling that my tasks overwhelm me

☐ Never

☐ Rarely
☐ Sometimes
☐ Often
☐ Very often

119.Too many tasks that I have to do

☐ Never
☐ Rarely
☐ Sometimes
☐ Often
☐ Very often

120.Situations in which I feel pleasure doing my work (school, apprenticeship, studies)

☐ Never
☐ Rarely
☐ Sometimes
☐ Often
☐ Very often

121.Times when so many difficulties accumulate that they are barely managed

☐ Never
☐ Rarely
☐ Sometimes
☐ Often
☐ Very often

German Version

Allgemeines

1. **Wann wurden Sie <u>geboren</u>?** |__|__| |__|__| 19|__|__|
   Tag Monat Jahr

2. **Sind Sie männlich oder weiblich?**
   ☐ männlich
   ☐ weiblich

3. **Welchen Familienstand haben Sie?**
   ☐ ledig
   ☐ verheiratet / eingetragene Lebenspartnerschaft
   ☐ geschieden / eingetragene Lebenspartnerschaft aufgehoben
   ☐ verwitwet

4. **Haben Sie Kinder? (Adoptiv- und Stiefkinder eingeschlossen)**
   ☐ Nein
   ☐ Ja

5. **Wie viele Kinder haben Sie?** |__|__| Kinder

6. **In welchem Jahr sind Ihre Kinder geboren?**

   Kind 1 Kind 2 Kind 3

   20|__|__| 20|__|__| 20|__|__|

Gesundheit

7. **Haben Sie jemals in den <u>letzten 12 Monaten</u> ein pfeifendes oder brummendes Geräusch in Ihrem Brustkorb gehört?**
   ☐ Nein
   ☐ Ja

8. **Hatten Sie dieses Pfeifen oder Brummen, wenn Sie nicht erkältet waren?**
   ☐ Nein
   ☐ Ja

9. Hatten Sie zu irgendeiner Zeit im Verlauf der <u>letzten 12 Monate</u> tagsüber einen Anfall von Kurzatmigkeit, der in Ruhe auftrat?

☐ Nein
☐ Ja

10. Hatten Sie in den <u>letzten 12 Monaten</u> einen Anfall von Kurzatmigkeit, der nach einer anstrengenden Tätigkeit auftrat (damit meinen wir nicht das normale „außer Atem sein“ nach sportlichen Aktivitäten)?

☐ Nein
☐ Ja

11. Sind Sie irgendwann in den <u>letzten 12 Monaten</u> durch einen Anfall von Luftnot aufgewacht?

☐ Nein
☐ Ja

12. Haben Sie an den meisten Tagen <u>für mindestens 3 Monate jährlich</u> Husten?

☐ Nein
☐ Ja

13. Falls ja, bestehen diese Beschwerden <u>seit mindestens 2 Jahren</u>?

☐ Nein
☐ Ja

14. Haben Sie an den meisten Tagen <u>für mindestens 3 Monate jährlich</u> Auswurf?

☐ Nein
☐ Ja

15. Falls ja, bestehen diese Beschwerden seit mindestens 2 Jahren?

☐ Nein
☐ Ja

16. Wurde bei Ihnen <u>von einem Arzt</u> schon einmal eine der folgenden Erkrankungen festgestellt?

Asthma

☐ Noch nie
☐ Einmal
☐ Mehrmals

Spastische / asthmatische Bronchitis

☐ Noch nie
☐ Einmal
☐ Mehrmals

Chronische Bronchitis

☐ Noch nie

☐ Einmal

☐ Mehrmals

17. Haben Sie jemals Asthma gehabt?

☐ Nein

☐ Ja

18. Wie alt waren Sie, als Sie Ihren ersten Asthmaanfall hatten? |__|__| Jahre

19. Wie alt waren Sie, als Sie Ihren letzten Asthmaanfall hatten? |__|__| Jahre

20. Hatten Sie in den letzten 12 Monaten einen Asthmaanfall?

☐ Nein

☐ Ja

21. Nehmen Sie gegenwärtig Medikamente gegen Asthma ein (einschließlich Inhalationen, Dosieraerosolen (Sprays) oder Tabletten)?

☐ Nein

☐ Ja

Wenn ja, welche:

22. Hatten Sie in den letzten 12 Monaten Probleme mit Niesanfällen oder einer laufenden, verstopften Nase, ohne erkältet zu sein?

☐ Nein

☐ Ja

23. Traten diese Nasenprobleme zusammen mit juckenden, tränenden Augen auf?

☐ Nein

☐ Ja

24. Haben Sie allergischen Schnupfen, zum Beispiel „Heuschnupfen"?

☐ Nein

☐ Ja

25. Hatten Sie in den letzten 12 Monaten allergischen Schnupfen, zum Beispiel „Heuschnupfen"?

☐ Nein

☐ Ja

26. Hat ein Arzt bei Ihnen schon einmal allergischen Schnupfen, zum Beispiel „Heuschnupfen"

festgestellt?

☐ Nein

☐ Ja

27. Wie alt waren Sie, als Sie allergischen Schnupfen oder Heuschnupfen zum ersten Mal hatten?

|__|__| Jahre

28. Sind Sie jemals in Ihrem Leben gegen eine Allergie desensibilisiert / hyposensibilisiert worden (dabei bekommt man zum Beispiel regelmäßig Spritzen oder legt sich regelmäßig eine Tablette unter die Zunge)?

☐ Nein

☐ Ja

☐ Weiß nicht

29. Hatten Sie irgendwann einmal Neurodermitis (atopisches Ekzem, endogenes Ekzem, atopische Dermatitis)?

☐ Nein

☐ Ja

30. Hat ein Arzt bei Ihnen schon einmal Neurodermitis (atopisches Ekzem, endogenes Ekzem, atopische Dermatitis) festgestellt?

☐ Nein

☐ Ja

31. Hatten Sie irgendwann einmal einen juckenden Hautausschlag, der stärker oder schwächer über mindestens 6 Monate auftrat?

☐ Nein

☐ Ja

32. Trat dieser juckende Hautausschlag bei Ihnen auch in den letzten 12 Monaten auf?

☐ Nein

☐ Ja

33. Betrifft dieser nur Ihre Hände?

☐ Nein

☐ Ja

34. Trat dieser juckende Hautausschlag bei Ihnen irgendwann einmal an einer der folgenden Körperstellen auf: in den Ellenbeugen oder Kniekehlen, an den Hand- oder Fußgelenken, im Gesicht, am Hals?

☐ Nein

☐ Ja

35. Haben Sie eine Nickelallergie (z.B. Ohrringe, Jeansknopf, Uhrenarmbänder)?

☐ Nein
☐ Ja

36. Hatten Sie jemals ein <u>Handekzem</u>?

☐ Nein
☐ Ja

37. Hatten Sie jemals ein Ekzem an Ihren <u>Handgelenken</u> oder <u>Unterarmen</u> (außer in den Ellenbeugen)?

☐ Nein
☐ Ja

38. Wann hatten Sie das letzte Mal ein Ekzem an Ihren Händen, Handgelenken oder Unterarmen?

Handekzem

☐ Ich habe es jetzt
☐ Nicht jetzt, aber in den letzten 3 Monaten
☐ Vor 3-12 Monaten
☐ Vor mehr als 12 Monaten
In welchem Jahr zum letzten Mal? |__|__|__|__|

Ekzem an Handgelenk / Unterarmen

☐ Ich habe es jetzt
☐ Nicht jetzt, aber in den letzten 3 Monaten
☐ Vor 3-12 Monaten
☐ Vor mehr als 12 Monaten
In welchem Jahr zum letzten Mal? |__|__|__|__|

39. Haben Sie bemerkt, dass Kontakt zu bestimmten Materialien, Chemikalien oder Sonstigem <u>an Ihrem Arbeitsplatz</u> Ihr Ekzem verschlimmert?

Handekzem

☐ Nein
☐ Ja
Wenn ja, was?
☐ Weiß nicht

Ekzem an Handgelenk / Unterarmen

☐ Nein
☐ Ja
Wenn ja, was?

☐ Weiß nicht

☐ Ich bin nicht berufstätig

40. Haben Sie bemerkt, dass Kontakt zu bestimmten Materialien, Chemikalien oder Sonstigem <u>außerhalb Ihres Arbeitsplatzes</u> Ihr Ekzem verschlimmert?

Handekzem

☐ Nein

☐ Ja

Wenn ja, was?

☐ Weiß nicht

Ekzem an Handgelenk / Unterarmen

☐ Nein

☐ Ja

Wenn ja, was?

☐ Weiß nicht

41. Verbessert sich Ihr Ekzem, wenn Sie <u>nicht an Ihrem normalen Arbeitsplatz</u> sind (z.B. an Wochenenden oder längeren Zeiten)?

Handekzem

☐ Nein

☐ Ja, manchmal

☐ Ja, normalerweise

☐ Weiß nicht

Ekzem an Handgelenk / Unterarmen

☐ Nein

☐ Ja, manchmal

☐ Ja, normalerweise

☐ Weiß nicht

☐ Ich bin nicht berufstätig

Wohnung

42. Seit wie vielen Jahren leben Sie in Ihrer jetzigen Wohnung / in Ihrem jetzigen Haus?

|__|__| Monate

oder

|__|__| Jahre

43. Welchen Fußboden hat das Zimmer, in dem Sie schlafen?

☐ Zugeschnittener Teppichboden, der die ganze Zimmerfläche bedeckt

☐ Läufer, Brücke, Teppich (z.B. Perserteppich)

☐ Aufwischbarer Belag

44. Haben Sie in den letzten 10 Jahren irgendeine der folgenden Maßnahmen durchgeführt, um einer Allergie vorzubeugen oder allergische Beschwerden zu verringern?

☐ Austausch eines Teppichs gegen einen Holzfußboden oder andere glatte Böden
wann (Jahr)? 20|__|__|

☐ Anschaffung eines antiallergischen Matratzenüberzugs (Milbendicht)
wann (Jahr)? 20|__|__|

☐ Abschaffung eines Haustiers
wann (Jahr)? 20|__|__|

☐ Keine dieser Maßnahmen

45. Waren jemals Schimmel oder Stockflecken auf irgendwelchen Oberflächen in Ihrem Heim außer auf Nahrungsmitteln? Mit Stockflecken meinen wir gelbliche oder bräunliche Verfärbung durch Feuchtigkeit.

Zur Zeit

☐ Nein
☐ Ja
☐ Weiß nicht

Innerhalb der letzten 10 Jahre

☐ Nein
☐ Ja
☐ Weiß nicht

Falls ja, in welchen Räumen ist / war der Schimmel vorzufinden?

Im Wohnzimmer

☐ Kein Schimmelbefall
☐ Kleiner als 0,5 m^2
☐ Zwischen 0,5 und 1 m^2
☐ Größer als 1 m^2

Im Schlafzimmer

☐ Kein Schimmelbefall
☐ Kleiner als 0,5 m^2
☐ Zwischen 0,5 und 1 m^2
☐ Größer als 1 m^2

Im Kinderzimmer

☐ Kein Schimmelbefall
☐ Kleiner als 0,5 m^2
☐ Zwischen 0,5 und 1 m^2
☐ Größer als 1 m^2

In der Küche

☐ Kein Schimmelbefall

☐ Kleiner als 0,5 m^2

☐ Zwischen 0,5 und 1 m^2

☐ Größer als 1 m^2

Im Bad

☐ Kein Schimmelbefall

☐ Kleiner als 0,5 m^2

☐ Zwischen 0,5 und 1 m^2

☐ Größer als 1 m^2

In einem anderen Raum

☐ Kein Schimmelbefall

☐ Kleiner als 0,5 m^2

☐ Zwischen 0,5 und 1 m^2

☐ Größer als 1 m^2

46. Mit welchen der folgenden Haustiere haben Sie zurzeit Kontakt?

In der eigenen Wohnung

☐ Mit keinen Haustieren

☐ Hund

☐ Katze

☐ Vogel

☐ Andere

Welche?

In einer anderen Wohnung

☐ Mit keinen Haustieren

☐ Hund

☐ Katze

☐ Vogel

☐ Andere

Welche?

47. Welche Ihrer Haustiere dürfen in Ihr Bett?

☐ Keine

☐ Hund

☐ Katze

☐ Andere

Welche?

48. Wie oft sind Sie <u>außerhalb Ihrer Berufstätigkeit</u> mit folgenden Tätigkeiten beschäftigt?

Putz- / Spültätigkeiten ohne Handschuhe

☐ Nie
☐ < 1 Tag pro Woche
☐ 1-3 Tage pro Woche
☐ ≥ 4 Tage pro Woche

Hausbau / Renovierung

☐ Nie
☐ < 1 Tag pro Woche
☐ 1-3 Tage pro Woche
☐ ≥ 4 Tage pro Woche

Garten / Landwirtschaft

☐ Nie
☐ < 1 Tag pro Woche
☐ 1-3 Tage pro Woche
☐ ≥ 4 Tage pro Woche

Sonstige Tätigkeiten, die durch Nässe, Chemikalien oder andere Faktoren hautbelastend sein könnten

☐ Nie
☐ < 1 Tag pro Woche
☐ 1-3 Tage pro Woche
☐ ≥ 4 Tage pro Woche

Welche?

49. Verwenden Sie Hautschutz- / Hautpflegemittel (Salben, Cremes usw.)?

☐ Nein
☐ Ja

50. Wie oft verwenden Sie diese zu Hause? (bitte nur eine Angabe machen)

ca. |__|__| Mal täglich
oder
ca. |__|__| Mal wöchentlich

51. Wie oft verwenden Sie diese bei der Arbeit? (bitte nur eine Angabe machen)

ca. |__|__| Mal täglich
oder
ca. |__|__| Mal wöchentlich
☐ Ich bin nicht berufstätig

52. An wie vielen Tagen pro Woche verwenden Sie die folgenden Sprays?

Möbelsprays

☐ Nie
☐ < 1 Tag pro Woche
☐ 1-3 Tage pro Woche
☐ ≥ 4 Tage pro Woche

Glasreinigungssprays (Fenster, Spiegel)

☐ Nie
☐ < 1 Tag pro Woche
☐ 1-3 Tage pro Woche
☐ ≥ 4 Tage pro Woche

Sprays für Teppiche, Bettvorleger, Läufer, Gardinen

☐ Nie
☐ < 1 Tag pro Woche
☐ 1-3 Tage pro Woche
☐ ≥ 4 Tage pro Woche

Backofensprays

☐ Nie
☐ < 1 Tag pro Woche
☐ 1-3 Tage pro Woche
☐ ≥ 4 Tage pro Woche

Bügelsprays

☐ Nie
☐ < 1 Tag pro Woche
☐ 1-3 Tage pro Woche
☐ ≥ 4 Tage pro Woche

Raumsprays

☐ Nie
☐ < 1 Tag pro Woche
☐ 1-3 Tage pro Woche
☐ ≥ 4 Tage pro Woche

Körperpflegespray / Deodorantspray

☐ Nie
☐ < 1 Tag pro Woche
☐ 1-3 Tage pro Woche
☐ ≥ 4 Tage pro Woche

Andere Sprays

☐ Nie
☐ < 1 Tag pro Woche
☐ 1-3 Tage pro Woche

☐ ≥ 4 Tage pro Woche
Welche?

53. Verwenden Sie Desinfektionsmittel?

☐ Nein
☐ Ja

54. Wie viele Stunden pro Tag verwenden Sie diese Desinfektionsmittel?

Zu Hause
☐ Nie
☐ < 1 Stunde pro Tag
☐ 1-3 Stunden pro Tag
☐ ≥ 4 Stunden pro Tag

Bei der Arbeit
☐ Nie
☐ < 1 Stunde pro Tag
☐ 1-3 Stunden pro Tag
☐ ≥ 4 Stunden pro Tag

☐ Ich bin nicht berufstätig

55. An wie vielen Tagen pro Woche (in der Arbeit oder zu Hause) verwenden Sie die folgenden Desinfektionsmethoden?

Sprühdesinfektion
☐ Nie
☐ < 1 Tag pro Woche
☐ 1-3 Tage pro Woche
☐ ≥ 4 Tage pro Woche

Mit der Hand mit Desinfektionsmitteln abwaschen
☐ Nie
☐ < 1 Tag pro Woche
☐ 1-3 Tage pro Woche
☐ ≥ 4 Tage pro Woche

Mit der Maschine desinfizieren
☐ Nie
☐ < 1 Tag pro Woche
☐ 1-3 Tage pro Woche
☐ ≥ 4 Tage pro Woche

Oberflächen mit Schwamm / Lappen mit Desinfektionsmittel reinigen
☐ Nie

☐ < 1 Tag pro Woche
☐ 1-3 Tage pro Woche
☐ ≥ 4 Tage pro Woche

Wischen oder Scheuern von Böden mit Desinfektionsmitteln

☐ Nie
☐ < 1 Tag pro Woche
☐ 1-3 Tage pro Woche
☐ ≥ 4 Tage pro Woche

Andere Methoden

☐ Nie
☐ < 1 Tag pro Woche
☐ 1-3 Tage pro Woche
☐ ≥ 4 Tage pro Woche

Welche?

Rauchen

56. Haben Sie schon einmal ein Jahr lang geraucht? „Ja“ bedeutet mindestens 20 Päckchen Zigaretten oder 360 g Tabak in Ihrem Leben oder ein Jahr lang mindestens eine Zigarette pro Tag oder eine Zigarre pro Woche.

☐ Nein
☐ Ja

57. Wie alt waren Sie, als Sie anfingen zu rauchen? |__|__| Jahre

58. Haben Sie innerhalb des letzten Monats geraucht?

☐ Nein
☐ Ja

59. Wie alt waren Sie, als Sie das Rauchen aufgegeben haben? |__|__| Jahre

60. Wie viel rauchen (bzw. rauchten) Sie durchschnittlich? |__|__| Zigaretten/Tag

61. Haben Sie schon einmal eine Wasserpfeife oder Shisha geraucht?

☐ Nein
☐ Ja

62. Haben Sie in den letzten 12 Monaten Wasserpfeife oder Shisha geraucht?

☐ Nein

☐ Ja

63. **Wenn Sie jetzt einmal an die letzten 30 Tage denken: An wie vielen Tagen haben Sie Wasserpfeife oder Shisha geraucht?** An |__|__| Tagen

64. **Haben Sie schon einmal E-Zigarette gedampft / geraucht?**
☐ Nein
☐ Ja

65. **Haben Sie in den letzten 12 Monaten E-Zigarette gedampft / geraucht?**
☐ Nein
☐ Ja

66. **Wenn Sie jetzt einmal an die letzten 30 Tage denken: Wie viel ml Liquid bzw. wie viele Züge dampfen / rauchen Sie mit der E-Zigarette durchschnittlich am Tag?**
|__|__|__| ml Liquid/Tag
oder
|__|__|__| Anzahl Züge/Tag

67. **Sind Sie regelmäßig in den <u>letzten 12 Monaten</u> Tabakrauch oder E-Zigaretten-Dampf Anderer ausgesetzt gewesen? Regelmäßig bedeutet: mindestens einmal an den meisten Tagen oder Nächten.**
☐ Nein
☐ Ja, nur Tabakrauch
☐ Ja, nur E-Zigaretten-Dampf
☐ Ja, Tabakrauch und E-Zigaretten-Dampf

68. **Bitte beschreiben Sie dies genauer: Wie viele Stunden sind Sie <u>pro Tag</u> Tabakrauch von anderen an den folgenden Orten ausgesetzt?**
Zu Hause
    |__|__| Stunden
    ☐ weniger als 1 Stunde
Am Arbeitsplatz
    |__|__| Stunden
    ☐ weniger als 1 Stunde
In Bars, Restaurants, Kinos oder ähnlichem
    |__|__| Stunden
    ☐ weniger als 1 Stunde
Im Auto
    |__|__| Stunden
    ☐ weniger als 1 Stunde

Andere Orte

|__|__| Stunden

☐ weniger als 1 Stunde

Arbeitssitation

69. Welchen beruflichen Ausbildungsabschluss haben Sie?

☐ Keinen beruflichen Abschluss und nicht in beruflicher Ausbildung

☐ Abgeschlossene beruflich-betriebliche Ausbildung (Lehre) bzw. beruflich-schulische Ausbildung (Berufsfachschule, Handelsschule)

☐ Abgeschlossene Ausbildung an einer Fachschule, Meister-, Technikerschule, Berufs- oder Fachakademie

☐ Fachhochschulabschluss

☐ Hochschulabschluss (Bachelor)

☐ Hochschulabschluss (Master, Diplom)

☐ Anderen beruflichen Abschluss

☐ Derzeit noch in beruflicher Ausbildung (Auszubildende/r, Lehrling, Berufsfach- oder Handelsschule)

☐ Student/in

☐ Sonstiges

Bitte angeben

70. Sind Sie zurzeit…?

☐ Auszubildende/r / Berufsschüler/in

☐ Student/in

☐ Angestellt

☐ Verbeamtet

☐ Selbstständig

☐ Arbeitslos und arbeitssuchend

☐ Aus gesundheitlichen Gründen nicht arbeitend

☐ Hausfrau / Hausmann (hauptberuflich)

☐ In Mutterschutz / Elternzeit oder sonstige Beurlaubung

☐ Sonstiges

Bitte angeben

71. Haben Sie innerhalb der letzten 10 Jahre irgendeine Arbeit / ein Praktikum für mindestens 1 Monat ausgeführt?

☐ Nein

☐ Ja

72. Welche Art von Arbeitsstellen und / oder Praktika hatten Sie in den letzten zehn Jahren?
Dabei ist jede Arbeit wichtig, die Sie für mindestens 1 Monat ausgeübt haben. Hierbei ist es egal, ob Sie diese Arbeit außer Haus oder zu Hause ausgeführt haben, Vollzeit oder Teilzeit, mit Lohn oder unentgeltlich oder als Selbstständiger (z.B. in einem Familienunternehmen).
Bitte geben Sie in dieser Frage Tätigkeiten nur dann an, wenn Sie diese mindestens 8 Stunden pro Woche ausgeführt haben.
Beginnen Sie bitte mit der letzten Arbeitsstelle.

☐ Ich habe keine Tätigkeit für mindestens 8 Stunden pro Woche ausgeführt

Tätigkeit 1

Tätigkeit / Beruf
Branche
Wann haben Sie mit dieser Tätigkeit begonnen?
|__|__|/|__|__|__|__| Monat/Jahr
Wenn zutreffend: Wann haben Sie mit dieser Tätigkeit aufgehört?
|__|__|/|__|__|__|__| Monat/Jahr
Wie viele Stunden pro Woche führ(t)en Sie diese Tätigkeit durch?
|__|__| Stunden

…

Tätigkeit 10

Tätigkeit / Beruf
Branche
Wann haben Sie mit dieser Tätigkeit begonnen?
|__|__|/|__|__|__|__| Monat/Jahr
Wenn zutreffend: Wann haben Sie mit dieser Tätigkeit aufgehört?
|__|__|/|__|__|__|__| Monat/Jahr
Wie viele Stunden pro Woche führ(t)en Sie diese Tätigkeit durch?
|__|__| Stunden

73. Waren Sie innerhalb der vergangenen 12 Monate bei Ihrer Arbeit folgenden Situationen ausgesetzt?

☐ Benachteiligung aufgrund Ihres Alters
☐ Benachteiligung aufgrund Ihrer Herkunft, ethnischen Zugehörigkeit oder Hautfarbe
☐ Benachteiligung aufgrund Ihrer Nationalität
☐ Benachteiligung aufgrund Ihres Geschlechts
☐ Benachteiligung aufgrund Ihrer Religion
☐ Benachteiligung aufgrund einer Behinderung
☐ Benachteiligung aufgrund Ihrer sexuellen Orientierung
☐ Ich war keiner dieser Situationen ausgesetzt

74. Sind Sie in den letzten 12 Monaten bei der Verrichtung Ihrer Arbeit mit folgenden Situationen konfrontiert worden?

☐ Körperliche Gewalt
☐ Sexuelle Belästigungen
☐ Mobbing / Schikanierung
☐ Ich war mit keiner dieser Situationen konfrontiert

75. Kommt es regelmäßig zu Änderungen Ihrer Arbeitszeiten? Falls ja, wie lange im Voraus wissen Sie normalerweise über diese Änderung Bescheid?

☐ Nein
☐ Ja, am selben Tag
☐ Ja, am Vortag
☐ Ja, wenige Tage im Voraus
☐ Ja, einige Tage im Voraus
☐ Ja, einige Wochen im Voraus
☐ Sonstiges
    Bitte angeben

76. Wie gut lassen sich Ihre Arbeitszeiten im Allgemeinen mit Ihren familiären oder sozialen Verpflichtungen außerhalb des Berufs vereinbaren?

☐ Sehr gut
☐ Gut
☐ Nicht sehr gut
☐ Überhaupt nicht

77. Wie oft ist es Ihnen in den letzten 12 Monaten passiert, dass Sie in Ihrer Freizeit gearbeitet haben, um die Arbeitsanforderungen zu erfüllen?

☐ Fast jeden Tag
☐ Ein- oder zweimal in der Woche
☐ Ein- oder zweimal im Monat
☐ Seltener
☐ Nie

78. Haben Sie durch eine Ihrer Tätigkeiten in den letzten 10 Jahren Niesanfälle oder eine laufende, verstopfte Nase bekommen, ohne erkältet zu sein?

☐ Nein
☐ Ja

79. Traten diese Nasenprobleme zusammen mit juckenden, tränenden Augen auf?

☐ Nein
☐ Ja

80. Haben Sie durch eine dieser Tätigkeiten Engegefühle in der Brust bzw. ein Pfeifen oder Brummen in der Brust bekommen?

☐ Nein
☐ Ja

81. Bei welcher dieser Tätigkeiten haben Sie eine dieser Beschwerden bekommen?

☐ Tätigkeit 1
☐ Tätigkeit 2
☐ Tätigkeit 3
☐ Tätigkeit 4
☐ Tätigkeit 5
☐ Tätigkeit 6
☐ Tätigkeit 7
☐ Tätigkeit 8
☐ Tätigkeit 9
☐ Tätigkeit 10

82. Mussten Sie eine dieser Tätigkeiten aufgrund der Beschwerden aufgeben?

☐ Nein
☐ Ja

83. Welche dieser Tätigkeiten musste aufgrund der Beschwerden aufgegeben werden?

☐ Tätigkeit 1
☐ Tätigkeit 2
☐ Tätigkeit 3
☐ Tätigkeit 4
☐ Tätigkeit 5
☐ Tätigkeit 6
☐ Tätigkeit 7
☐ Tätigkeit 8
☐ Tätigkeit 9
☐ Tätigkeit 10

84. Tragen Sie bei der Arbeit Handschuhe?

☐ Nein
☐ Manchmal
☐ Ja

85. Wie lange tragen Sie die Handschuhe während Ihrer Arbeitszeit?

☐ In weniger als 25% der Arbeitszeit
☐ Zwischen 25% und 50% der Arbeitszeit
☐ Mehr als 50% der Arbeitszeit

86. Wurde bei Ihnen schon mal eine Berufskrankheiten-Anzeige gestellt?
☐ Nein
☐ Ja

87. Aufgrund welcher Beschwerden wurde die Berufskrankheiten-Anzeige gestellt?
☐ Aufgrund von Atemwegsbeschwerden
☐ Aufgrund von Hautbeschwerden
☐ Aufgrund anderer Beschwerden, bitte angeben
Welche?

Sport

88. Wie <u>häufig</u> und <u>wo</u> treiben Sie gewöhnlich Sport, so dass Sie außer Atem geraten oder schwitzen?
Sport im Freien
☐ Nie
☐ Weniger als einmal pro Monat
☐ Einmal pro Monat
☐ Einmal pro Woche
☐ 2-3 mal pro Woche
☐ 4-6 mal pro Woche
☐ Jeden Tag
Sport in der Halle / in geschlossenen Räumen
☐ Nie
☐ Weniger als einmal pro Monat
☐ Einmal pro Monat
☐ Einmal pro Woche
☐ 2-3 mal pro Woche
☐ 4-6 mal pro Woche
☐ Jeden Tag

89. Wie viele <u>Stunden in der Woche</u> und <u>wo</u> treiben Sie gewöhnlich Sport, so dass Sie außer Atem geraten oder schwitzen?
Sport im Freien
☐ Keine

☐ Ca. 0,5 Stunden
☐ Ca. 1 Stunde
☐ Ca. 2-3 Stunden
☐ Ca. 4-6 Stunden
☐ 7 Stunden und mehr

Sport in der Halle / in geschlossenen Räumen

☐ Keine
☐ Ca. 0,5 Stunden
☐ Ca. 1 Stunde
☐ Ca. 2-3 Stunden
☐ Ca. 4-6 Stunden
☐ 7 Stunden und mehr

90. Welche Sportarten betreiben Sie?

☐ Laufen, Ballspiele, Radfahren, Inline-Skates
☐ Schwimmen
☐ Gemischte Belastung (z.B. Fitness-Studio)
☐ Sonstiges

Bitte angeben

91. Wie viele Stunden verbringen Sie in der Freizeit…

…pro Tag am Computer / Spielekonsole / Smartphone / Tablet?

|__|__| Stunden
☐ weniger als 1 Stunde

…pro Tag vor dem Fernseher (auch Videos / DVDs / Streamen von Sendungen)?

|__|__| Stunden
☐ weniger als 1 Stunde

92. Wie viele Stunden verbringen Sie in Ihrer Arbeit…

…pro Tag am Computer / Smartphone / Tablet?

|__|__| Stunden
☐ weniger als 1 Stunde

Körperliche Entwicklung

93. Wie groß sind Sie? |__|__|__| cm

94. Wie viel wiegen Sie? |__|__|__| kg

95. Nehmen Sie zurzeit die Antibabypille oder andere hormonelle Verhütungsmittel ein?

☐ Nein

☐ Ja

96. Seit wie vielen Jahren wenden Sie das Präparat an? seit ca. |__|__| Jahren

97. Wie oft waren Sie schwanger?

|__| Mal

☐Noch nie

Belastungssituationen

Wie oft fühlten Sie sich im Verlauf der <u>letzten 2 Wochen</u> durch die folgenden Beschwerden beeinträchtigt?

98. 1.5 Wenig Interesse oder Freude an Ihren Tätigkeiten

☐ Überhaupt nicht

☐ An einzelnen Tagen

☐ An mehr als der Hälfte der Tage

☐ Beinahe jeden Tag

1.6

99. 1.7 Niedergeschlagenheit, Schwermut oder Hoffnungslosigkeit?

☐ Überhaupt nicht

☐ An einzelnen Tagen

☐ An mehr als der Hälfte der Tage

1.8 ☐ Beinahe jeden Tag

Die folgenden Fragen sollen Sie danach beurteilen, ob Sie die darin angesprochenen Erfahrungen nie, selten, manchmal, häufig oder sehr häufig gemacht bzw. erlebt haben. Denken Sie bitte an das letzte Jahr und versuchen Sie sich daran zu erinnern, wie oft das jeweilige Ereignis eingetreten ist.

100. Aufschieben dringend benötigter Erholung

☐ Nie

☐ Selten

☐ Manchmal

☐ Häufig

☐ Sehr häufig

101. Situationen, in denen ich mich anstrengen muss, das Vertrauen anderer zu gewinnen

☐ Nie
☐ Selten
☐ Manchmal
☐ Häufig
☐ Sehr häufig

102. Zu wenig Zeit, um meine täglichen Aufgaben zu erledigen

☐ Nie
☐ Selten
☐ Manchmal
☐ Häufig
☐ Sehr häufig

103. Befriedigung durch die Arbeit (Schule, Ausbildung, Studium), die ich täglich zu erledigen habe

☐ Nie
☐ Selten
☐ Manchmal
☐ Häufig
☐ Sehr häufig

104. Situationen, in denen ich mich um eine gute Beziehung zu anderen bemühen muss

☐ Nie
☐ Selten
☐ Manchmal
☐ Häufig
☐ Sehr häufig

105. Zeiten, in denen ich Aufgaben zu erledigen habe, die ich nicht gern mache

☐ Nie
☐ Selten
☐ Manchmal
☐ Häufig
☐ Sehr häufig

106. Ich habe Aufgaben zu erledigen, bei denen ich unter kritischer Beobachtung stehe

☐ Nie
☐ Selten
☐ Manchmal

- ☐ Häufig
- ☐ Sehr häufig

107. Erfahrung, dass alles zu viel ist, was ich zu tun habe

- ☐ Nie
- ☐ Selten
- ☐ Manchmal
- ☐ Häufig
- ☐ Sehr häufig

108. Ich habe Arbeiten zu erledigen, bei denen ich andere nicht enttäuschen darf

- ☐ Nie
- ☐ Selten
- ☐ Manchmal
- ☐ Häufig
- ☐ Sehr häufig

109. Kontakte mit anderen Personen, bei denen ich einen guten Eindruck hinterlassen muss

- ☐ Nie
- ☐ Selten
- ☐ Manchmal
- ☐ Häufig
- ☐ Sehr häufig

110. Überforderung durch verschiedenartige Aufgaben, die ich zu erledigen habe

- ☐ Nie
- ☐ Selten
- ☐ Manchmal
- ☐ Häufig
- ☐ Sehr häufig

111. Situationen, in denen es ganz allein von mir abhängt, ob ein Kontakt zu einem anderen Menschen zufriedenstellend verläuft

- ☐ Nie
- ☐ Selten
- ☐ Manchmal
- ☐ Häufig
- ☐ Sehr häufig

112. Ich habe Aufgaben zu erfüllen, bei denen ich mich bewähren muss

- ☐ Nie
- ☐ Selten
- ☐ Manchmal
- ☐ Häufig
- ☐ Sehr häufig

113. Negative Einstellung zu den Arbeiten, die ich täglich zu erledigen habe

- ☐ Nie
- ☐ Selten
- ☐ Manchmal
- ☐ Häufig
- ☐ Sehr häufig

114. Zu viele Verpflichtungen, die ich unbedingt erfüllen muss

- ☐ Nie
- ☐ Selten
- ☐ Manchmal
- ☐ Häufig
- ☐ Sehr häufig

115. Situationen, in denen ich mich anstrengen muss, anderen zu gefallen

- ☐ Nie
- ☐ Selten
- ☐ Manchmal
- ☐ Häufig
- ☐ Sehr häufig

116. Wunsch, meinen jetzigen Beruf (Schule, Ausbildung, Studium) zu wechseln

- ☐ Nie
- ☐ Selten
- ☐ Manchmal
- ☐ Häufig
- ☐ Sehr häufig

117. Ich muss Aufgaben erfüllen, die mit hohen Erwartungen verbunden sind

- ☐ Nie
- ☐ Selten

- ☐ Manchmal
- ☐ Häufig
- ☐ Sehr häufig

118. Gefühl, dass mir meine Aufgaben über den Kopf wachsen

- ☐ Nie
- ☐ Selten
- ☐ Manchmal
- ☐ Häufig
- ☐ Sehr häufig

119. Zu viele Aufgaben, die ich zu erledigen habe

- ☐ Nie
- ☐ Selten
- ☐ Manchmal
- ☐ Häufig
- ☐ Sehr häufig

120. Situationen, in denen ich spüre, dass ich meine Arbeit (Schule, Ausbildung, Studium) gerne mache

- ☐ Nie
- ☐ Selten
- ☐ Manchmal
- ☐ Häufig
- ☐ Sehr häufig

121. Zeiten, in denen sich Schwierigkeiten so häufen, dass sie kaum zu bewältigen sind

- ☐ Nie
- ☐ Selten
- ☐ Manchmal
- ☐ Häufig
- ☐ Sehr häufig
